# A combined approach for individualized lymphadenectomy in gastric cancer patients

**DOI:** 10.3389/pore.2023.1611270

**Published:** 2023-06-29

**Authors:** Zsolt Varga, Adrienn Bíró, Miklós Török, Dezső Tóth

**Affiliations:** ^1^ Department of Surgery, University of Debrecen, Debrecen, Hungary; ^2^ Department of Surgery, Moritz Kaposi General Hospital, Kaposvár, Hungary; ^3^ Department of Pathology, University of Debrecen, Debrecen, Hungary

**Keywords:** gastric cancer, surgery, lymphadenectomy, sentinel, Maruyama

## Abstract

**Introduction:** Gastric cancer ranks as the fifth most common cancer globally. The presence of lymph node metastasis is a significant prognostic factor influencing survival. Postoperative morbidity and nodal staging accuracy are heavily affected by the extent of lymph node dissection. Our study aimed to explore the potential integration of two contemporary methods, sentinel node navigation surgery (SNNS) and the Maruyama Computer Program (MCP), to improve the accuracy of nodal staging.

**Materials and methods:** We conducted a prospective data collection involving patients with gastric adenocarcinoma from 2008 to 2018 at the Department of Surgery, University of Debrecen, Hungary. Data from 100 consecutive patients were collected. The primary and secondary endpoints included evaluating the rate of node-negative patients and the diagnostic accuracy of our combined approach.

**Results:** Sentinel node mapping was successful in 97 out of 100 patients. We found that using the threshold value of the Maruyama Index (MI) ≥ 28, all metastatic stations of sentinel-node-negative patients could be identified. Our method achieved 100% sensitivity and negative predictive value, with a specificity of 60.42% (95% CI = 46.31%–72.98%).

**Discussion:** The combined application of SNNS and MCP has proven to be an effective diagnostic technique in the synergistic approach for identifying metastasis-positive lymph node stations. Despite its limitations, this combination may assist clinicians in customizing lymphadenectomy for gastric cancer patients.

## Introduction

According to the recent GLOBOCAN 2020 estimation, gastric cancer is the fifth most common cancer worldwide [1]. The diagnosis is often made at an advanced stage, resulting in a high mortality rate. One of the most important prognostic factors after curative resection in patients with gastric adenocarcinoma is the presence of lymph node metastases.

In 1881, Theodor Billroth (1829–1894) performed the first successful gastric resection for gastric cancer. Despite scientific and technological advancements, including the development of the multimodal treatment approach, resection still remains at the forefront of curative management in gastric cancer [2]. The clinical stage determines the treatment approach since a stage-adapted, individualized treatment is crucial to achieve optimal oncological outcomes.

Surgery aims to establish local control through free surgical resection margins and the clearance of regional lymph nodes. Though, a significant proportion of patients have no nodal involvement after R0 resection. Postoperative morbidity and the accuracy of nodal staging are heavily influenced by the extent of lymph node dissection, although the optimal extent of lymph node dissection has been debated over the last decades. Insufficient lymphadenectomy may result in the understaging and undertreatment of a patient. Conversely, unnecessary lymph node dissection may have higher rates of postoperative complications [3–5].

Conventional preoperative imaging techniques, such as computed tomography (CT), magnetic resonance imaging (MRI), endoscopic ultrasound (EUS), and positron emission tomography (PET), provide an accurate T and M stage, but there is significant uncertainty regarding the N stage [6]. These techniques predominantly depend on size criteria for the detection of lymph node metastasis. However, metastatic lymph nodes that do not result in significant enlargement could be overlooked, as the resolution provided by CT, MRI, and PET scans might not be adequate to detect such small metastatic lymph nodes [7]. PET scans identify metabolic activity, but not all metastatic lymph nodes in gastric cancer display increased metabolic activity. Given the relatively poor spatial resolution, which makes distinguishing nodes from the primary tumor itself challenging, and the fact that inflammation can also result in higher metabolic activity, the utility of PET scans in this context may be limited [8]. EUS primarily assesses perigastric lymph nodes, and thus, may not detect skipping metastases that have spread directly to the D2 compartment, bypassing the perigastric area. In a study, the incidence of such metastases was found to be 4.8% among the overall gastric cancer population [9]. The differentiation between benign and malignant lymphadenopathy is merely based on morphological characteristics, which include size (greater than 5 mm), shape (round), echo pattern (hypoechoic), and border (smooth) [10].

In a comparative study, the detection rate of lymph node metastasis by CT scan was 71.7%, with a sensitivity of 44.6% and a specificity of 85.4%. The performance of endoscopic ultrasonography was similar, providing a detection rate of 70.4%, a sensitivity of 19.3%, and a specificity of 96.3% [11].

In 1973, the Japanese Research Society for Gastric Cancer described the lymphatic drainage pattern of the stomach in the first edition of their manual. They identified 16 unique lymph node stations based on anatomical locations and introduced a system for quantifying the extent of lymphadenectomy, namely, D1, D2, and D3. Since its inception, this guideline has undergone numerous revisions [12].

The sentinel lymph node (SLN) is defined as the first node to receive lymphatic flow from a tumor, theoretically representing the status of the other regional lymph nodes. Sentinel node navigation surgery (SNNS) is a type of surgical technique in which the SLN is removed and examined for the presence of cancer cells [13]. During SNNS, a tracer substance is injected near the tumor which then travels through the lymph vessels and ends up in the sentinel node(s). In gastric cancer surgery, various tracers have been used: blue dye, indocyanine green (ICG), radiocolloids, and their combinations [14]. The extent of lymph node dissection might be determined according to the status of the SLN. If the sentinel lymph node is free of metastases, a gastrectomy, and D2 lymph node dissection may not be necessary. This promising approach may lead to a lesser extent of resection and lymph node dissection, resulting in organ preservation, faster postoperative recovery, and better quality of life (QoL) without compromising oncological safety [14]. It can also increase the surgeons’ awareness of critical lymph node stations, thereby improving the effectiveness of lymphadenectomy for each patient. Unfortunately, metastatic lymph nodes are not always stained by the tracer, and conversely, stained nodes are not necessarily metastatic. In practice, there are several challenges, particularly when dealing with gastric cancer. The process of identifying and sampling the sentinel lymph node(s) is technically demanding, requiring careful procedural execution and pathological analysis. Errors or variations in the surgeon’s technique can lead to missed sentinel nodes [15]. Furthermore, lymphatic drainage patterns can significantly differ between individuals, and even within different regions of the same tumor [16]. This variability can sometimes lead to unpredictable sentinel nodes, which could potentially be missed. If cancer has spread to a lymph node and has blocked the lymphatic drainage pathway, the tracer used to identify the sentinel lymph node may be diverted to a different lymph node, leading to a false-negative result [17]. On top of that, micrometastases or isolated tumor cells might not be detected during routine pathological examination of the sentinel lymph node, also potentially leading to a false-negative result [18]. The concept of sentinel (lymphatic) basin dissection was introduced to address many of the limitations associated with previous techniques. This approach was first introduced by Miwa et al. in 2003 [15]. Their technique entailed not just the removal of the stained sentinel node, but also the associated nodal compartment (basin), thereby achieving an accuracy of 98.0%.

There are two large-scale ongoing randomized clinical trials to determine the role of SNNS in gastric cancer surgery. The SENORITA trial failed to show non-inferiority of SNNS compared with laparoscopic standard gastrectomy in terms of 3-year disease-free survival (DFS) [19]. Still, the 5-year disease-free survival and overall survival did not reveal the statistical difference between the two groups [20]. This concept has yet to be proven in a broader clinical setting. It is worth noting, that its’ strict inclusion criteria might compromise its general applicability to the Western population.

The Maruyama Computer Program (MCP), developed by Keiichi Maruyama and released in 1989, is a tool designed to estimate the likelihood of lymph node involvement in stations No. 1–16, based on several prognostic factors [21]. These factors include seven preoperative variables: sex, age, tumor type, depth of invasion, tumor location, primary tumor diameter, and histological type. After providing these elements, the software is computing a numerical value for each station. MCP was initially validated in a Japanese patient cohort, where it achieved a predictive accuracy of lymph node involvement in 94% of cases [22]. The program’s precision was further enhanced from 66% to 93% through the application of an artificial neural network [23].

In our previous work, we proved the high reliability of MCP, achieving a sensitivity of 90.2%, a specificity of 63.3%, and an overall accuracy of 78.4%. We also demonstrated that MCP’s predictive capabilities for lymph node metastases were superior to those of standard pre-operative imaging techniques [24].

The MCP has traditionally served as an effective tool for projecting long-term oncological results. The term “Maruyama Index” (MI) was coined by Hundahl et al. [25] in the aftermath of the Intergroup 0116 Trial. They conducted a blinded re-evaluation of the Dutch D1-D2 trial using autopsy findings and demonstrated that surgeries characterized as “low MI” (which entails not leaving behind any lymphatic stations with high MI) had a more beneficial impact on survival compared to surgeries guided solely by D-levels [26].

Our objective was to explore the potential integration of the MCP and SNNS to improve the accuracy of detecting nodal involvement and to identify a possible method that could make tailored lymphadenectomy safer. We aimed to evaluate the individual efficacy of these techniques and determine MI values that could optimally supplement SNNS in a synergetic manner.

## Materials and methods

**FIGURE 1:**
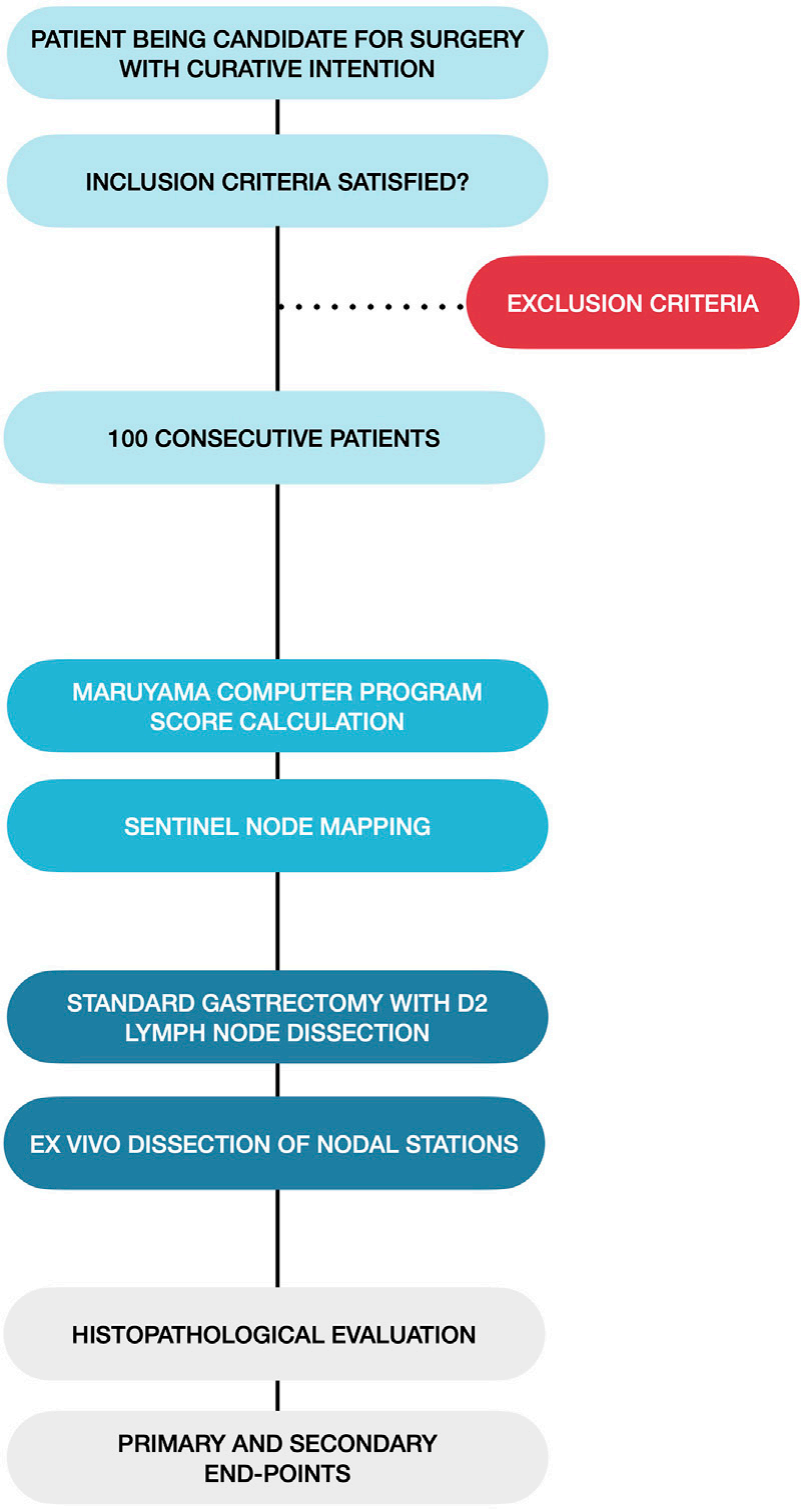
Flowchart of the patient management and data collection process.

A prospective data collection of patients operated on with a curative intention of gastric adenocarcinoma was conducted from 2008 until 2018 (Figure 1). It was a single-center study, with every procedure being performed at the Department of Surgery, University of Debrecen, Hungary. Adult patients (>18 years of age) who were willing and able to provide written informed consent were included. Another requirement was that the patient had a histologically confirmed adenocarcinoma in the stomach sampled by preoperative esophagogastroduodenoscopy (EGD). Complete oncological staging (including chest-, abdomen-, and pelvis CT scan) and evaluation of fitness for surgery were required.

Exclusion criteria were gross (macroscopic) serosal or nearby organ invasion, history of gastric resection, cancer of the gastric cardia (<5 cm distance from the gastroesophageal junction), and distant metastases. Patients with macroscopic serosal invasion had only a peritoneal lavage at the index procedure, without any resection. The cytological examination was carried out later as the peritoneal fluid sample was processed. Positive cytology was taken as verification of peritoneal dissemination and M1 stage disease. These patients were treated accordingly.

Before 2018, neoadjuvant treatment was not frequently used for non-cardia gastric cancer in Hungary. However, as this approach became routine, we chose to finish our data and patient collection to prevent any systematic bias that could be introduced with the inclusion of patients undergoing neoadjuvant therapy. There is considerable controversy about the degree and significance of nodal regression after neoadjuvant treatment [27–29]. Our study population could be one of the last cohorts of patients with locally advanced gastric cancer, who did not receive any preoperative oncological treatment, in which the unharmed lymphatic drainage of gastric cancer could be observed.

Our primary and secondary end-points were the assessment of the rate of node-negative patients and the accuracy of our combined technique.

We collected data for 100 consecutive patients. Patient information, such as age, gender, comorbidities, location, histological type, and clinical and pathological stage of the tumor was recorded. Meanwhile, three different (sixth, seventh, and eighth) editions of the UICC TNM classification of malignant tumors had been in use. These editions have well-known differentiating factors in the staging of gastric cancer. To consolidate our findings, all previous documents were revised by a medical oncologist and a pathologist and reported according to the eighth edition.

**FIGURE 2:**
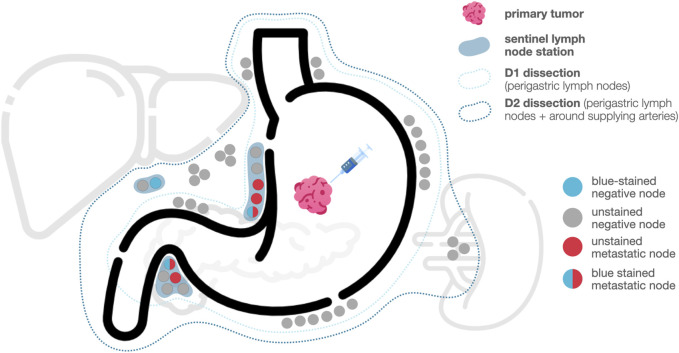
Types of nodal involvement in sentinel node navigation surgery.

Before the surgical intervention, the Maruyama Computer Program (MCP; WinEstimate v2.5 Gastric cancer diagnosis and treatment. An interactive training program. Windows Version. CD-ROM) was used to assess the probability of lymph node metastasis in each nodal station. The software-generated Maruyama Index (MI) was noted for each lymphatic station. During the procedure, peritumoral–subserosal or submucosal injection of 2 mL blue dye (Bleu Patente V 2.5/100 mg, Guerbet, France) was used for sentinel node mapping (Figure 2). After the injection, a 10-min waiting period was given to allow lymphatic drainage. Then a conventional (total, subtotal, or distal) open or laparoscopic gastrectomy with standard D2 lymphadenectomy was performed. The blue-stained sentinel lymph nodes were assessed with intraoperative frozen sections. The D3 compartment (stations 13–16) and station 10 nodes were sampled only if clinical suspicion was high for metastasis.

After completing the surgery, *ex vivo* dissection and categorization of the lymph nodes were carried out. The anatomical position of each lymph node station was labeled according to the Japanese Classification of Gastric Carcinoma (JCGC). The presence of a (blue-colored) sentinel node in a station was marked for the pathologist.

All dissected lymph nodes were sent for postoperative histopathologic evaluation, following a protocol similar to the one used in the SENORITA trial [30]. Nodes from the blue-stained stations were examined for micrometastases using 0.2 mm sections. Otherwise, a slice was made at 2-mm intervals parallel to the long axis. Hematoxylin and eosin (HE) staining and pan-cytokeratin immunohistochemistry examinations were performed (Monoclonal Mouse Anti-Human Cytokeratin, Clones AE1/AE3; DAKO, Denmark).

All patient data collected during this study was recorded in a standalone offline Microsoft Excel database.

Continuous variables were tested for normality by the Shapiro-Wilk test. Data with normal distribution are shown as mean ± standard deviation or medians with a range for non-Gaussian distribution. They were analyzed by the Student’s t-test and ANOVA test, or an appropriate non-parametric method (Mann–Whitney U-test or Kruskal–Wallis).

Categorical variables shown as counts and percentages were analyzed by Fisher’s exact test. The level of statistical significance was set at *p* < 0.05 for all analyses.

The sentinel node detection rate was defined as the number of patients with a successful marking of the sentinel lymph node, divided by the total number of patients. The sensitivity and specificity in sentinel node mapping were calculated according to the number of patients with metastatic lymph nodes in the sentinel station, and the number of patients with metastatic lymph nodes.

All statistical analyses were performed using IBM SPSS Statistics for Macintosh, Version 28.0 (Released 2021. Armonk, NY: IBM Corp).

## Results

There were 53 women and 47 men among the enrolled patients. Their median age was 67 (48–82) years. The median BMI was 23.0 (17.5–36.0) kg/m^2^. The tumor was located in the upper third in 13 cases (13%), in the middle third in 21 (21%) cases, and in the lower third in 66 cases (66%). 38 patients had a moderately differentiated adenocarcinoma, 31 patients had signet-ring cell carcinoma, 27 patients had poorly differentiated adenocarcinoma, and 4 patients had well-differentiated adenocarcinoma. Table 1 shows the descriptive data of these patients. We performed a total gastrectomy on 39 patients, and a distal gastrectomy on 61 patients. The laparoscopic technique was used in 27 cases (3 total gastrectomies, 24 laparoscopic-assisted distal gastrectomies). Despite gross serosal or nearby organ invasion being in the exclusion criteria, the histopathology report showed 30 patients with pT4a stage. After the revision of these patients’ data, we decided not to exclude their results from the analysis as they could prove beneficial in understanding the behavior of advanced gastric cancer. The total number of removed lymph nodes was 2083, and the median number of dissected nodes from a patient was 20 (range: 9–42).

**TABLE 1:** Descriptive data of patients included in the study.

Factors	Results
Number of patients (n)	100
Gender	
Female	53
Male	47
Median age (range; in years)	67 (48–82)
Median body mass index (BMI; kg/m^2^)	23 (17.5–36.0)
Tumor location	
Upper third	13
Middle third	21
Lower third	66
Histological subtype	
Signet-ring cell carcinoma	31
Poorly differentiated adenocarcinoma	27
Moderately differentiated adenocarcinoma	38
Well-differentiated adenocarcinoma	4
Surgical technique	
Open surgery	73
Laparoscopic surgery	27
Pathological T-stage	
T1a	8
T1b	13
T2	16
T3	33
T4a	30
Pathological N-stage	
N0	48
N1	19
N2	17
N3	16
Total number of dissected lymph nodes	2083
Median number of dissected lymph nodes (range)	20 (9–42)

The sentinel node mapping was successful in 97 of the 100 patients, resulting in a 97.0% detection rate. The median number of blue-stained (sentinel) nodes was 7 (range: 0–19) and the median number of labeled stations was 2 (0–6). The number of labeled nodes was not associated with the BMI of the patient (*p* = 0.705), location (*p* = 0.254), the histological subtype of the tumor (*p* = 0.891), pT stage (*p* = 0.227), or operative (open or laparoscopic) technique (*p* = 0.393). The location of the sentinel nodes was in the D1 compartment in 80 cases (82.47%), both in the D1 and D2 compartments in 16 cases (16.50%), and exclusively in the D2 compartment in 1 case (1.03%).

We explored the connection between the pT stage of the tumor, the success rate of sentinel node mapping, and the presence of lymph node metastases.

Among the 21 patients with pT1 stage tumors, most (18 patients, or 85.71%) had no lymph node metastases. The remaining three patients had metastases confined to the stations containing the sentinel nodes, with no involvement of any other lymph node stations. In two cases, sentinel node mapping was not successful; however, these patients were found to be without lymph node involvement (pN0 stage).

In the group of patients with pT2 tumors (totaling 16), more than half (nine patients, or 56.25%) had no lymph node metastases. When lymph node metastases were present, they were always located within the sentinel nodes. Sentinel node mapping was successfully carried out for all patients in this group, identifying seven patients with lymph node metastases. In each case, the metastases were exclusively situated within the blue-stained D1 station.

Among the 33 patients with pT3 stage tumors, 14 (42.42%) were found to have no lymph node involvement. Of the remaining 19 patients with lymph node involvement, all but one had metastases within the sentinel nodes. There was a single instance of a non-sentinel station showing positivity, and one patient had no labeled station.

As for the patients with pT4a stage tumors (*n* = 30), seven cases (23.33%) had no lymph node metastases. When lymph node metastases were present, they were almost always (21 out of 23 cases, or 91.30%) confined to the same station as the sentinel node.

The overall sensitivity of sentinel node mapping in patients with successful labeling (*n* = 97) was 94.23% (95% CI = 84.36%–98.43%) with 100% specificity (95% CI = 92.73%–100%), *p* < 0.001 (Table 2). This level of sensitivity is considered excellent, however, relying solely on this technique would have resulted in overlooking nodal metastases in three patients. Of the 48 sentinel node-negative patients, all 3 patients had macrometastasis, and no micrometastasis was found in the sentinel nodes.

**TABLE 2:** The ability of SNNS to find metastatic lymph node stations.

Patients	pN+	pN−
SLNB negative	3	45
SLNB not informative/positive	49	3
Total	52	48
**Identification of metastatic lymph node stations by SLNB (not informative/positive)**	**Value**	**95% CI**
Sensitivity	94.23%	84.36%–98.43%
Specificity	93.75%	83.16%–97.85%
Positive predictive value	94.23%	84.36%–98.43%
Negative predictive value	93.75%	83.16%–97.85%
		*p* < 0.001

SLNB, sentinel lymph node biopsy; pN+, positive pathologic lymph node staging; pN−, negative pathologic lymph node staging; 95% CI, 95% confidence interval.

To improve this 93.75% negative predictive value, we combined the results of the sentinel node mapping technique with the Maruyama Computer Program.

In our previous research, we used receiver operating characteristic (ROC) curve analysis to fine-tune the cut-off value for the Maruyama Index [24]. In this current investigation, we aimed to achieve a sensitivity of 100% (thus eliminating any false negative cases) using the combined technique. This goal, however, led to a reduction in specificity (resulting in more false positives). Upon evaluating the results of the MCP predictions and histological reports, we found that an MI cut-off value of 28 or higher could deliver the desired level of sensitivity. Using the MCP alone with this threshold, we correctly identified 44 of the 52 patients with at least one node-positive station. The sensitivity of the MCP alone was 84.62% (95% CI = 72.48%–91.99%), and its specificity was 52.08% (95% CI = 38.33%–65.53%), Table 3. The positive predictive value was 65.57% (95% CI = 53.73%–75.91%), and the negative predictive value was 75.76% (95% CI = 58.98%–87.17%).

**TABLE 3:** The ability of MCP to find metastatic lymph node stations.

Patients	pN+	pN−
MI < 28	8	25
MI ≥ 28	44	23
Total	52	48
**Identification of metastatic lymph node stations by MI ≥ 28**	**Value**	**95% CI**
Sensitivity	84.62%	72.48%–91.99%
Specificity	52.08%	38.33%–65.53%
Positive predictive value	65.57%	53.73%–75.91%
Negative predictive value	75.76%	58.98%–87.17%
		*p* < 0.001

MI, maruyama index; pN+, positive pathologic lymph node staging; pN−, negative pathologic lymph node staging; 95% CI, 95% confidence interval.

**FIGURE 3:**
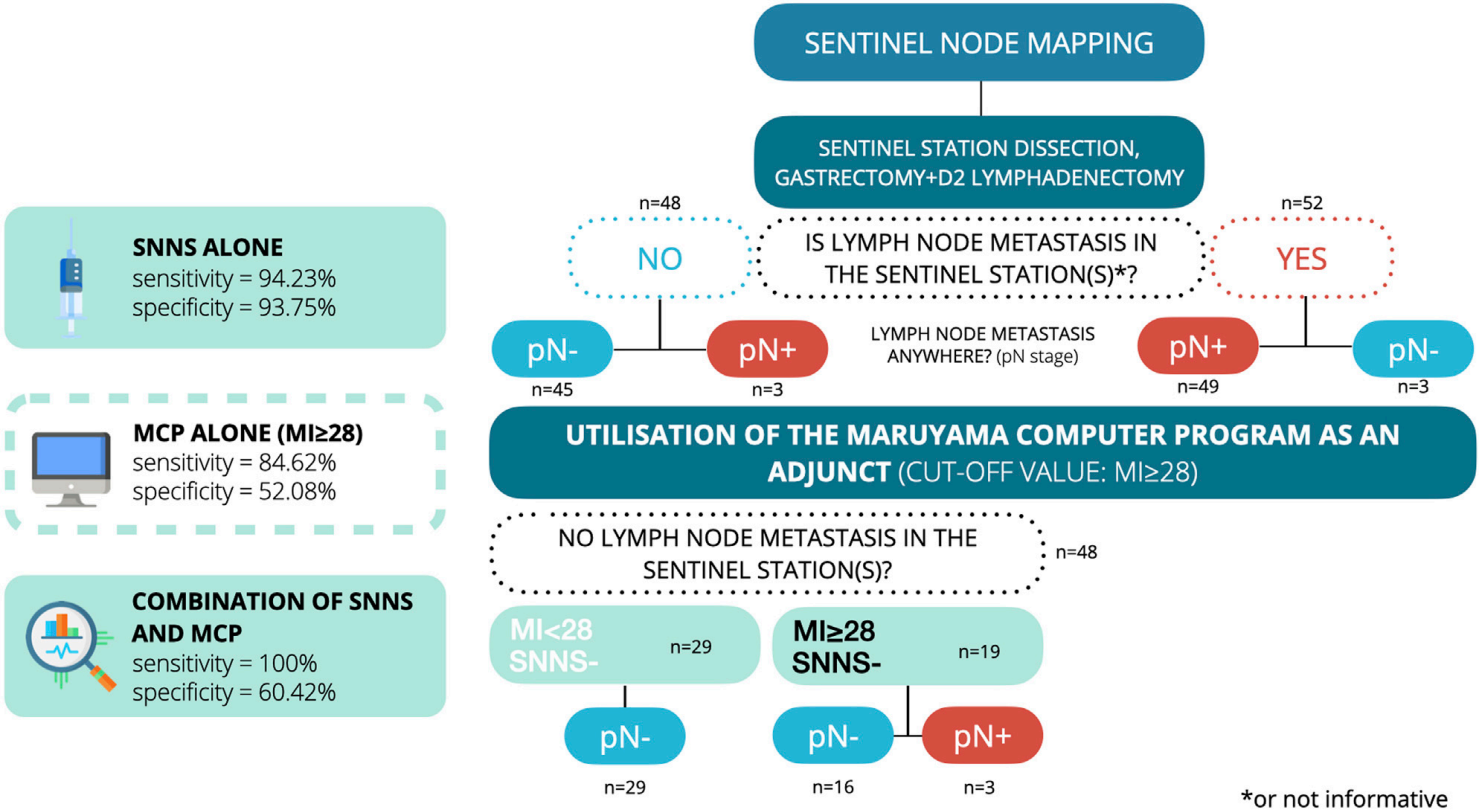
Flowchart for the diagnostic accuracy of the combined technique.

In our effort to integrate the two techniques, for patients who were sentinel node negative, we set a threshold for the Maruyama Index at ≥28 (Figure 3). This allowed us to identify all metastatic stations. With this approach, we were able to attain a sensitivity and negative predictive value of 100% (95% CI = 93.12%–100% and 88.30%–100%, respectively), as well as a specificity of 60.42% (95% CI = 46.31%–72.98%). The positive predictive value was determined to be 73.42% (95% CI = 61.95%–82.15%) (Table 4). The median number of additional stations (those that were non-stained and had a Maruyama Index ≥28) that needed to be dissected was 1, with a range of 1–5.

**TABLE 4:** The ability of the combination of SNNS and MCP to find metastatic lymph node stations.

Patients	pN+	pN−
SLNB negative and MI < 28	0	29
SLNB not informative/positive and/or MI ≥ 28	52	19
Total	52	48
**Identification of metastatic lymph node stations by SLNB (not informative/positive) and/or MI ≥ 28**	**Value**	**95% CI**
Sensitivity	100%	93.12%–100%
Specificity	60.42%	46.31%–72.98%
Positive predictive value	73.24%	61.95%–82.15%
Negative predictive value	100%	88.30%–100%
		*p* < 0.001

SLNB, sentinel lymph node biopsy; MI, maruyama index; pN+, positive pathologic lymph node staging; pN−, negative pathologic lymph node staging; 95% CI, 95% confidence interval.

30 of the 79 (37.97%) pT2-4a stage patients were node-negative and had unnecessary complete D2 lymphadenectomy. All node-positive patients and their metastatic stations were correctly identified with 100% accuracy (refer to Table 4) using the combination method. Using this synergetic approach, these 30 patients would have a median number of 4 stations (range: 1–7) removed. We found no side effects in the blue dye mapping.

## Discussion

The predicted prognosis of any gastrointestinal cancer is significantly influenced by the presence of lymph node metastases. A sufficient level of lymphadenectomy helps to avoid both stage migration and the undertreatment of patients, however unnecessary lymph node dissection can result in a higher risk for iatrogenic injuries and higher postoperative morbidity. Accurate prediction of the presence and location of lymph node metastasis is necessary to reduce the extent of lymphadenectomy, without undermining the oncological safety.

The JCOG0302 trial in Japan had to be discontinued due to the unexpectedly high false-negative rate of 46.4% [31]. The study began in May 2004 but was halted in September 2005 after enrolling 440 patients. The explanation for this termination was the discovery of false negative results in 13 patients. The main reason for this unreliability was the single-plane frozen section. To avoid these issues, we adopted a protocol similar to that used in the Korean SENORITA trial [30]. According to this protocol, nodes thicker than 4 mm are sliced at 2 mm intervals along the long axis to ensure that macrometastasis is not overlooked. The results of the intraoperative frozen section correlated with the postoperative pathological findings in all the cases, as in our previous study [32]. In a previous study of ours, submucosal and subserosal marking methods were proven to be equivalent in detection rate. The sensitivity and specificity were 100% in the submucosal group, and 100% and 92.3% in the subserosal group, respectively [33].

For the safe implementation of nodal staging based on SN mapping results, particularly in an intraoperative setting, it is crucial to accurately detect lymph node metastases. Compared to HE and IHC staining, molecular biology methods like reverse transcription polymerase chain reaction (RT-PCR) can provide more accuracy by allowing examination of the entire lymph node. Multiple RT-PCR methods have proven suitable for intraoperative detection of (micro) metastases, but their application can be time-consuming. The one-step nucleic acid amplification (OSNA) assay emerges as a quicker, appealing alternative diagnostic tool for identifying SN metastases in gastric cancer patients [34].

In gastric adenocarcinoma, the lymphatic drainage of the tumor can be complicated. A significant amount of patient data has been collected over the past decades to find consistency among the results. The Maruyama Computer Program was invented based on this predictability. It is usually utilized in advanced gastric cancer to explore potential lymph node metastases preoperatively. Sentinel node navigation surgery is another tool that helps us to find potential metastatic lymph nodes. By combining these two approaches, we synthesized an efficient way to detect those stations and identify those patients who need extended lymphadenectomy with 100% sensitivity and 60.42% specificity. The exceptionally high level of sensitivity in our model needs to be further validated by independent studies.

Several limitations of our study require further discussion. The selection criteria for patients could potentially lead to a selection bias. It is particularly true for pT4a stage cases, since some of them may not be included in our investigation by the macroscopic serosal invasion.

Some well-established predictive factors for lymph node metastasis, such as grade, tumor size, or lymphovascular invasion were not included in the analysis. This is because the route of lymphatic drainage is not influenced by them, thus the number and location of the involved stations are independent of their existence.

In an era of routine perioperative chemotherapy for cT2/N+ gastric cancer, our findings emphasize a personalized approach. This enables us to pay more attention to potentially affected stations, irrespective of the level of lymph node dissection. The data collection for evaluating the survival outcomes is in progress.

We demonstrated that the flaws of each of these modern techniques could be overcome by their combination, making it a powerful method. We are not advocating for the omission of preoperative nodal clinical staging or the standard D2 lymphadenectomy. Instead, our goal is to draw attention to a potentially beneficial tool that can enhance surgeons’ awareness of critical lymph node stations. Nevertheless, in selected cases, the extent of lymphadenectomy could potentially be safely reduced using this method. Despite these limitations, this synergic approach of sentinel node mapping with the Maruyama Computer Program may help clinicians tailor lymphadenectomy for gastric cancer patients.

## Data Availability

The raw data supporting the conclusion of this article will be made available by the authors, without undue reservation.

## Abbreviations

EGD, esophagogastroduodenoscopy; HE, hematoxylin and eosin; ICG, indocyanine green; JCGC, Japanese Classification of Gastric Carcinoma; MCP, Maruyama Computer Program; MI, Maruyama Index; OSNA, one-step nucleic acid amplification; pN+, positive pathologic lymph node staging; pN−, negative pathologic lymph node staging; QoL, quality of life; RT-PCR, reverse transcription polymerase chain; SLN, sentinel lymph node; SLNB, sentinel lymph node biopsy; SNNS, sentinel node navigation surgery; 95% CI, 95% confidence interval.

## References

[1] Sung H Ferlay J Siegel RL Laversanne M Soerjomataram I Jemal A Global cancer Statistics 2020: Globocan estimates of incidence and mortality worldwide for 36 cancers in 185 countries. CA Cancer J Clin (2021) 71(3):209–49. 10.3322/caac.21660 33538338

[2] Eom SS Choi W Eom BW Park SH Kim SJ Kim YI A comprehensive and comparative review of global gastric cancer treatment guidelines. J Gastric Cancer (2022) 22(1):3–23. 10.5230/jgc.2022.22.e10 35425651 PMC8980601

[3] Hartgrink HH van de Velde CJ Putter H Bonenkamp JJ Klein Kranenbarg E Songun I Extended lymph node dissection for gastric cancer: Who may benefit? Final results of the randomized Dutch gastric cancer group trial. J Clin Oncol (2004) 22(11):2069–77. 10.1200/JCO.2004.08.026 15082726

[4] Degiuli M Reddavid R Tomatis M Ponti A Morino M Sasako M D2 dissection improves disease-specific survival in advanced gastric cancer patients: 15-Year follow-up results of the Italian gastric cancer study group D1 versus D2 randomised controlled trial. Eur J Cancer (2021) 150:10–22. 10.1016/j.ejca.2021.03.031 33887514

[5] Songun I Putter H Kranenbarg EM Sasako M van de Velde CJ. Surgical treatment of gastric cancer: 15-Year follow-up results of the randomised nationwide Dutch D1d2 trial. Lancet Oncol (2010) 11(5):439–49. 10.1016/S1470-2045(10)70070-X 20409751

[6] Habermann CR Weiss F Riecken R Honarpisheh H Bohnacker S Staedtler C Preoperative staging of gastric adenocarcinoma: Comparison of helical ct and endoscopic us. Radiology (2004) 230(2):465–71. 10.1148/radiol.2302020828 14752188

[7] Kim HJ Kim AY Oh ST Kim JS Kim KW Kim PN Gastric cancer staging at multi-detector row ct gastrography: Comparison of transverse and volumetric ct scanning. Radiology (2005) 236(3):879–85. 10.1148/radiol.2363041101 16020558

[8] Findlay JM Antonowicz S Segaran A El Kafsi J Zhang A Bradley KM Routinely staging gastric cancer with (18)F-fdg pet-ct detects additional metastases and predicts early recurrence and death after surgery. Eur Radiol (2019) 29(5):2490–8. 10.1007/s00330-018-5904-2 30643947 PMC6443603

[9] Choi YY An JY Guner A Kang DR Cho I Kwon IG Skip lymph node metastasis in gastric cancer: Is it skipping or skipped? Gastric Cancer (2016) 19(1):206–15. 10.1007/s10120-015-0472-5 25708370

[10] Faige DO. Eus in patients with benign and malignant lymphadenopathy. Gastrointest Endosc (2001) 53(6):593–8. 10.1067/mge.2001.114060 11323584

[11] Hwang SW Lee DH Lee SH Park YS Hwang JH Kim JW Preoperative staging of gastric cancer by endoscopic ultrasonography and multidetector-row computed tomography. J Gastroenterol Hepatol (2010) 25(3):512–8. 10.1111/j.1440-1746.2009.06106.x 20370729

[12] Japanese Gastric Cancer A. Japanese gastric cancer treatment guidelines 2018 (5th edition). Gastric Cancer (2021) 24(1):1–21. 10.1007/s10120-020-01042-y 32060757 PMC7790804

[13] Ohdaira H Nimura H Mitsumori N Takahashi N Kashiwagi H Yanaga K. Validity of modified gastrectomy combined with sentinel node navigation surgery for early gastric cancer. Gastric Cancer (2007) 10(2):117–22. 10.1007/s10120-007-0419-6 17577622

[14] Wei J Bu Z. Sentinel lymph node detection for gastric cancer: Promise or pitfall? Surg Oncol (2020) 33:1–6. 10.1016/j.suronc.2019.12.005 31885358

[15] Miwa K Kinami S Taniguchi K Fushida S Fujimura T Nonomura A. Mapping sentinel nodes in patients with early-stage gastric carcinoma. Br J Surg (2003) 90(2):178–82. 10.1002/bjs.4031 12555293

[16] Lee SE Lee JH Ryu KW Cho SJ Lee JY Kim CG Sentinel node mapping and skip metastases in patients with early gastric cancer. Ann Surg Oncol (2009) 16(3):603–8. 10.1245/s10434-008-0283-6 19127361

[17] Liu JY Deng JY Zhang NN Liu HF Sun WL He WT Clinical significance of skip lymph-node metastasis in Pn1 gastric-cancer patients after curative surgery. Gastroenterol Rep (Oxf) (2019) 7(3):193–8. 10.1093/gastro/goz008 31217983 PMC6573797

[18] Jeuck TL Wittekind C. Gastric carcinoma: Stage migration by immunohistochemically detected lymph node micrometastases. Gastric Cancer (2015) 18(1):100–8. 10.1007/s10120-014-0352-4 24550066

[19] Ryu KW Kim YW Min JS An JY Yoon HM Eom BW Laparoscopic sentinel node navigation surgery versus laparoscopic standard gastrectomy with lymph node dissection in early gastric cancer: Final three-year survival results of multicenter randomized controlled phase iii trial (senorita trial). J Clin Oncol (2020) 38(15):4510. 10.1200/JCO.2020.38.15_suppl.4510

[20] Hur H Lee YJ Kim Y-W Min J-S Yoon HM An JY Clinical efficacy of laparoscopic sentinel node navigation surgery for early gastric cancer: Five-year results of senorita trial. J Clin Oncol (2022) 40(16):4050. 10.1200/JCO.2022.40.16_suppl.4050

[21] Kampschoer GH Maruyama K van de Velde CJ Sasako M Kinoshita T Okabayashi K. Computer analysis in making preoperative decisions: A rational approach to lymph node dissection in gastric cancer patients. Br J Surg (1989) 76(9):905–8. 10.1002/bjs.1800760910 2804584

[22] Maruyama K Gunven P Okabayashi K Sasako M Kinoshita T. Lymph node metastases of gastric cancer. General pattern in 1931 patients. Ann Surg (1989) 210(5):596–602. 10.1097/00000658-198911000-00005 2818028 PMC1357792

[23] Bollschweiler EH Monig SP Hensler K Baldus SE Maruyama K Holscher AH. Artificial neural network for prediction of lymph node metastases in gastric cancer: A phase ii diagnostic study. Ann Surg Oncol (2004) 11(5):506–11. 10.1245/ASO.2004.04.018 15123460

[24] Toth D Torok M Kincses Z Damjanovich L. Prospective, comparative study for the evaluation of lymph node involvement in gastric cancer: Maruyama computer program versus sentinel lymph node biopsy. Gastric Cancer (2013) 16(2):201–7. 10.1007/s10120-012-0170-5 22740059

[25] Hundahl SA Macdonald JS Benedetti J Fitzsimmons T Southwest Oncology Group and the Gastric Intergroup. Surgical treatment variation in a prospective, randomized trial of chemoradiotherapy in gastric cancer: The effect of undertreatment. Ann Surg Oncol (2002) 9(3):278–86. 10.1007/BF02573066 11923135

[26] Peeters KC Hundahl SA Kranenbarg EK Hartgrink H van de Velde CJ. Low Maruyama index surgery for gastric cancer: Blinded reanalysis of the Dutch D1-D2 trial. World J Surg (2005) 29(12):1576–84. 10.1007/s00268-005-7907-9 16317484

[27] Athauda A Nankivell M Langer R Pritchard S Langley RE von Loga K Pathological regression of primary tumour and metastatic lymph nodes following chemotherapy in resectable og cancer: Pooled analysis of two trials. Br J Cancer (2023) 128(11):2036–43. 10.1038/s41416-023-02217-x 36966233 PMC10206103

[28] Zhu YL Sun YK Xue XM Yue JY Yang L Xue LY. Unnecessity of lymph node regression evaluation for predicting gastric adenocarcinoma outcome after neoadjuvant chemotherapy. World J Gastrointest Oncol (2019) 11(1):48–58. 10.4251/wjgo.v11.i1.48 30984350 PMC6451926

[29] Pereira MA Ramos M Dias AR Cardili L Ribeiro RRE Charruf AZ Lymph node regression after neoadjuvant chemotherapy: A predictor of survival in gastric cancer. J Surg Oncol (2020) 121(5):795–803. 10.1002/jso.25785 31773740

[30] Park JY Kim YW Ryu KW Nam BH Lee YJ Jeong SH Assessment of laparoscopic stomach preserving surgery with sentinel basin dissection versus standard gastrectomy with lymphadenectomy in early gastric cancer-a multicenter randomized phase iii clinical trial (senorita trial) protocol. BMC Cancer (2016) 16:340. 10.1186/s12885-016-2336-8 27246120 PMC4886393

[31] Miyashiro I Hiratsuka M Sasako M Sano T Mizusawa J Nakamura K High false-negative proportion of intraoperative histological examination as a serious problem for clinical application of sentinel node biopsy for early gastric cancer: Final results of the Japan clinical oncology group multicenter trial Jcog0302. Gastric Cancer (2014) 17(2):316–23. 10.1007/s10120-013-0285-3 23933782

[32] Toth D Kincses Z Plosz J Torok M Kovacs I Kiss C Value of sentinel lymph node mapping using a blue dye-only method in gastric cancer: A single-center experience from north-east Hungary. Gastric Cancer (2011) 14(4):360–4. 10.1007/s10120-011-0048-y 21538019

[33] Toth D Kathy S Csoban T Kincses Z Torok M Plosz J Gyomortumorok őrszemnyirokcsomó-jelölésének prospektív, összehasonlító vizsgálata – submucosus kontra subserosus jelölés. Magy Seb (2012) 65(1):3–8. 10.1556/MaSeb.65.2012.1.1 22343099

[34] Shimada A Takeuchi H Nishi T Mayanagi S Fukuda K Suda K Utility of the one-step nucleic acid amplification assay in sentinel node mapping for early gastric cancer patients. Gastric Cancer (2020) 23(3):418–25. 10.1007/s10120-019-01016-9 31667687

